# The differential sensitivity of the hypothalamic–hypophysial–ovarian axis to 5‐hydroxytryptophan alters the secretion of estradiol

**DOI:** 10.1113/EP091158

**Published:** 2023-12-08

**Authors:** Juana Monroy, Omar D. Cortés, Roberto Domínguez, María Eugenia Mendoza‐Garrido, Eloir Gallegos, Mario Cárdenas, Andrés Aragón, María E. Ayala

**Affiliations:** ^1^ Laboratorio de Pubertad, Unidad de Investigación en Biología de la Reproducción, Facultad de Estudios Superiores Zaragoza Universidad Nacional Autónoma de México Mexico City Mexico; ^2^ Departamento de Fisiología, Biofísica y Neurociencias, Centro de Investigación y Estudios Avanzados (CINVESTAV) Instituto Politécnico Nacional Mexico City Mexico; ^3^ Departamento de Biología de la Reproducción Instituto Nacional de Ciencias Médicas y Nutrición Salvador Zubirán Mexico City Mexico; ^4^ Laboratorio de Gametos y Desarrollo Tecnológico, Facultad de Estudios Superiores Iztacala Universidad Nacional Autónoma de México Tlalnepantla State of México Mexico

**Keywords:** 5‐hydroxytryptophan, estradiol, gonadotrophins, hypophysis, hypothalamus, ovary, serotonin

## Abstract

Serotonin [5‐hydroxytryptamine (5‐HT)] modulates ovarian function. The precursor of 5‐HT, 5‐hydroxytryptophan (5‐HTP), has been used to treat depression. However, the effects of 5‐HTP on ovarian and reproductive physiology remain unknown. In this research, we analysed the impact of 5‐HTP on the monoaminergic system and its interactions with the reproductive axis and ovarian estradiol secretion when administered by distinct routes. Female rats 30 days of age were injected with 5‐HTP i.p. (100 mg/kg), into the ovarian bursa (1.5 µg/40 µL) or into the median raphe nucleus (20 µg/2.5 µL) and were killed 60 or 120 min after injection. As controls, we used rats of the same age injected with vehicle (0.9% NaCl). Monoamine, gonadotrophin and steroid ovarian hormone concentrations were measured. The injection of 5‐HTP either i.p. or directly into the ovarian bursa increased the concentrations of 5‐HT and the metabolite 5‐hydroxyindole‐3‐acetic acid in the ovary. For both routes of administration, the serum concentration of estradiol increased. After i.p. injection of 5‐HTP, the concentrations of luteinizing hormone were decreased and follicle‐stimulating hormone increased after 120 min. Micro‐injection of 5‐HTP into the median raphe nucleus increased the concentrations of 5‐HT in the anterior hypothalamus and dopamine in the medial hypothalamus after 120 min. Our results suggest that the administration of 5‐HTP either i.p. or directly into the ovarian bursa enhances ovarian estradiol secretion.

## Abstract (highlights)

- **What is the central question of this study?**
  Does the route of administration of 5‐hydroxytryptophan affect the synthesis of steroid sex hormones in prepubertal female rats?
- **What is the main finding and its importance?**
  The injection of 5‐hydroxytryptophan either i.p. or directly into the ovarian bursa in prepubertal female rats stimulated the serotoninergic system of the reproductive axis. In turn, this increased the secretion of estradiol, a gonadal hormone that regulates sexual maturation and several physiological processes in females.

## 1 INTRODUCTION

Serotonin [5‐hydroxytryptamine (5‐HT)] is involved in the regulation of a wide variety of physiological functions, including sensory and motor functions, memory, mood and reproductive hormone secretion. It has also been implicated in the aetiology of disorders such as anxiety, depression and sleep disorders (Jørgensen, 2007; Maffei, 2020; Moran et al., 2013; Terranova et al., 1990).

5‐Hydroxytryptophan (5‐HTP) is an intermediate molecule in the synthesis of 5‐HT and is used as a supplement to enhance the levels of 5‐HT in the treatment of several diseases related to serotonergic imbalance, such as depression (Jacobsen et al., 2016; Javelle et al., 2020). l‐Tryptophan is an essential amino acid; it is obtained from the diet because it is not synthesized by mammals (Church et al., 2020). 5‐Hydroxytryptamine is produced in the CNS, in the raphe nuclei of the brainstem (Jørgensen, 2007). It is also synthesized in peripheral organs, such as the gastrointestinal tract (Jones et al., 1980) and gonads (Amireault & Dubé, 2005; Dubé & Amireault, 2007). l‐Tryptophan is transformed by the action of the enzyme tryptophan hydroxylase (TPH) to form 5‐HTP, which is transformed into 5‐HT by the enzyme l‐aromatic decarboxylase (Hensler, 2012). Tryptophan hydroxylase has two isoforms: TPH1, which is found in several peripheral tissues and the pineal gland; and TPH2, which is a brain‐specific isoform expressed in serotoninergic neurons (Matthes & Bader, 2018). l‐Aromatic decarboxylase also catalyses the transformation of 3,4‐dihydroxyphenylalanine to dopamine (DA), which is converted into noradrenaline (NA) (Groaz et al., 2020). Both TPH and l‐aromatic decarboxylase have been described in the CNS (Hensler, 2012), the hypophysis (Carvajal et al., 1991; Payette et al., 1985; Vanhatalo et al., 1995) and the ovary (Amireault & Dubé, 2005; Dubé & Amireault, 2007; Nikishin et al., 2019). 5‐Hydroxytryptamine is catabolized by monoamine oxidase into 5‐hydroxyindole‐3‐acetic acid (5‐HIAA) after its reuptake into the presynaptic neuron. The concentration of 5‐HIAA in the brain is a measure of the turnover of 5‐HT (Hensler, 2012).

In the brain, the neurons that synthesize 5‐HT are organized into groups that are located in the brainstem and are designated as B1–B9. The dorsal raphe nucleus (DRN) corresponds to the B6 and B7 groups and median raphe nucleus (MRN) to the B5 and B8 groups. Serotonergic fibres that innervate different forebrain structures are projected principally from the DRN and MRN regions and are the main sources of 5‐HT for different hypothalamic nuclei (Hensler, 2012). The caudal serotonergic nuclei, the raphe magnus (B3), obscurus (B2) and pallidus (B1 and B4), project fibres to the spinal cord (Jacobs & Azmitia, 1992). In the hypophysis, the sources of 5‐HT are fibres of the MRN and platelets (Mezey et al., 1984; Shannon & Moore, 1987), whereas in the ovary, the sources of 5‐HT are platelets and mast cells, although it is also produced locally by granulosa cells (Amireault & Dubé, 2005; Nikishin et al., 2018, 2019). In the ovarian follicle, enzymes involved in 5‐HT synthesis, metabolism and transport are found (Nikishin et al., 2018, 2019). When 5‐hydroxytryptophan is added to follicles in vitro, very little 5‐HT is formed. Therefore, it is proposed that 5‐HT is not synthesized within the follicle itself but that it originates from external sources through reuptake or is synthesized within the gonad through the cooperation of different cell types (Nikishin et al., 2018).

5‐Hydroxytryptamine participates in the endocrine regulation of reproduction at three levels of the female reproductive axis: the hypothalamus, the hypophysis and the ovary. In the hypothalamus, 5‐HT produced by neurons arising from the MRN modulates the secretion of gonadotrophin‐releasing hormone (GnRH; Morello et al., 1990), which in turn stimulates the synthesis and release of gonadotrophins and directly regulates the production of steroid hormones in the ovary (Gallegos et al., 2022; Moran et al., 2013).

However, controversy exists regarding the involvement of serotonergic innervation of the hypothalamus from the MRN in the regulation of gonadotrophin secretion by the hypophysis. The 5‐HT originating in the MRN could have an inhibitory (Morello et al., 1989, 1990) or stimulatory (Meyer, 1978) effect or might have no effect on the secretion of luteinizing hormone (LH; Barofsky, 1975). Previously, we showed that in 30‐day‐old female rats, the micro‐injection of 5,6‐dihydroxytryamine, a selective neurotoxin of the serotonergic fibres, into the MRN does not modify the secretion of LH (Monroy et al., 2003). Therefore, the role of serotonergic innervation from the MRN to the hypothalamus in gonadotrophin secretion and control of steroidogenesis in the ovary remains to be established.

In the ovary in vitro, the secretion of steroid hormones is regulated by 5‐HT (Bódis et al., 1992; Graveleau et al., 2000; Tanaka et al., 1993; Terranova et al., 1990). For instance, in luteal cells of cows, activation of 5‐HT_1_ and 5‐HT_2_ receptors stimulated the synthesis of progesterone (Battista & Condon, 1986), whereas blocking of 5‐HT_2_ receptors in the follicles of rats (Tanaka et al., 1993) or the 5‐HT_1_ receptors in pre‐ovulatory follicles of hamsters (Terranova et al., 1990) decreased the production of estradiol. In the prepuberal rat, the injection of 5‐HT into the ovarian bursa resulted in reduced serum concentrations of estradiol. This event was considered to be an indicator of the participation of 5‐HT in the regulation of estradiol secretion by the ovary (Moran et al., 2013).

Oestrogen plays a key role in the regulation of several physiological processes in women, such as growth and maturation of the breasts, vasodilatation, vasoprotection, neuroprotection, metabolism, bone mineralization, behaviour and reproduction (Boon et al., 2010). The effects of administration of 5‐HTP on the hypothalamic–pituitary–ovarian axis have not been studied fully. One area of the utmost importance to understand the role of 5‐HTP in the reproductive axis is recognition of the effects of the route of administration of 5‐HTP on the production of steroid hormones.

A decrease in brain 5‐HT production is associated with anxiety, depression, sleep disorders and eating disorders (Maffei, 2020). Treatment with 5‐HTP offers an option for patients with these conditions (Bruni et al., 2004; Cross et al., 2011; Ryan et al., 1992), because it provides a natural means to increase brain 5‐HT concentrations (Haberzettl et al., 2013). Currently, there is no available information regarding the side effects of 5‐HTP treatment in children and adolescents or its potential effects on ovarian function.

The purpose of this study was to investigate the role of 5‐HTP in production of the hormones regulating the hypothalamic–hypophysial–ovarian axis of prepubertal female rats. To achieve our objective, we used rats 30 days of age, which were treated with 5‐HTP by the following distinct routes: i.p., into the ovarian bursa or into the MRN.

## 2 MATERIALS AND METHODS

### 2.1 Ethical approval

All experiments were carried out in strict accordance with the Mexican Law of Animal Treatment and Protection Guidelines and the specifications of the Mexican Official Standard NOM‐062‐ZOO‐1999 for the production, care and use of laboratory animals. The animals were maintained under care following protocols approved by the Committee of Facultad de Estudios Superiores Zaragoza (letter no. FESZ/DEPI/CE/001/21). All possible efforts were made to minimize the number of animals used and the extent of suffering endured by the animals.

### 2.2 Animals

Thirty‐day‐old female rats of the CII‐ZV strain from our own breeding stock were used in this study. The animals weighed 75–90 g at the time of treatment or surgery. The rats were maintained in controlled conditions for light (lights on from 05.00 to 19.00 h) and temperature (22 ± 2°C), with free access to food (Purina, SA de CV, Ciudad de México, Mexico) and tap water. Ten rats per experimental group and time of killing were used in each experiment.

### 2.3 Experimental design

**FIGURE 1:**
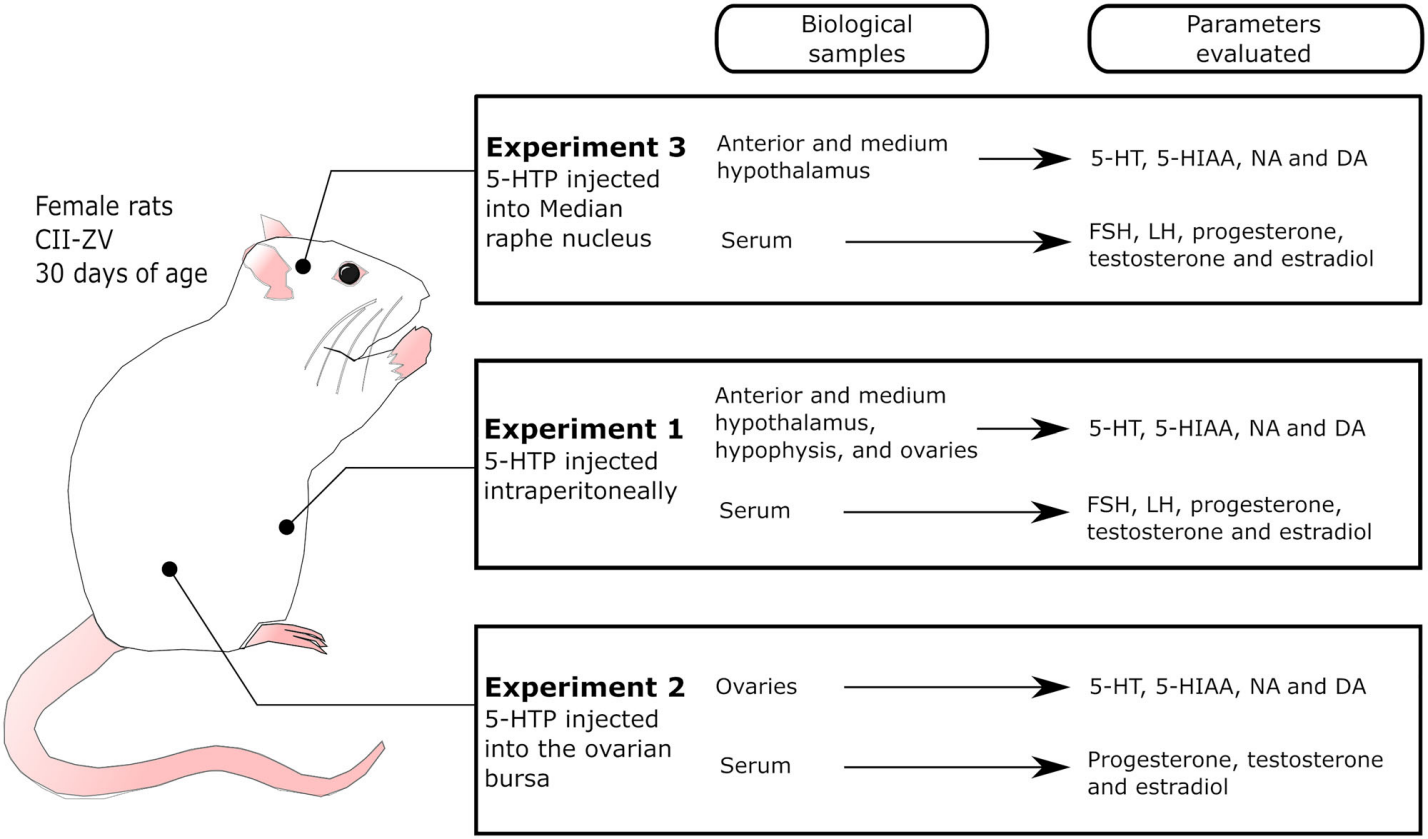
Schematic representation of the experimental design. Three independent experiments were realized. In each experiment, 5‐HTP was administered by a distinct route. In each experiment, the groups of rats were killed at 60 or 120 min after the treatment with 5‐HTP or vehicle (*n* = 8–10 rats per group and time). Statistical comparisons were made among treatments of the same experiment. Abbreviations: DA, dopamine; FSH, follicle stimulating hormone; 5‐HIAA, 5‐hydroxyindole‐3‐acetic acid; 5‐HT, 5‐hydroxytryptamine; 5‐HTP, 5‐hydroxytryptophan; LH, luteinizing hormone; NA, noradrenaline.

In order to examine the effects of 5‐HTP on reproductive axis functioning, three experiments were carried out. The first experiment was intended to analyse the systemic effects of 5‐HTP administration. The purpose of the second experiment was to examine the effects of 5‐HTP administered directly into the ovarian bursa. The third experiment was intended to analyse the effects of 5‐HTP administered into the MRN. The biological samples and parameters evaluated in each experiment are depicted in Figure 1.

Rats were allocated randomly to one of three groups. In the absolute control (AC) group, the rats were left untouched; this control was common to all experiments. We also included a vehicle (Vh; 0.9% NaCl) control group, in addition to a group treated with 5‐HTP. All animals in the different experiments were killed 60 or 120 min after treatment or micro‐injection. These time points were selected considering that 1 h after oral administration of 5‐HTP, the concentration of 5‐HT increased, and this effect was maintained during the second hour after the initiation of treatment (Das et al., 2004).

#### 2.3.1 Experiment 1: Systemic administration of 5‐HTP

Initially, we examined the effects of 5‐HTP on functioning of the reproductive axis. This experiment was intended to analyse the systemic effects of 5‐HTP on gonadotrophin and steroid hormone secretion. Rats (*n* = 8−10 per group) were injected i.p. with a dose of 100 mg/kg of 5‐HTP (Sigma, St Louis, MO, USA). The Vh group was injected with an equivalent volume of saline (0.9%). The dose of 5‐HTP was selected on the basis of previously published work (Gartside et al., 1992) indicating that 100 mg/kg of 5‐HTP can enhance neuronal 5‐HT synthesis. Neurotransmitters (5‐HT, 5‐HIAA, DA and NA) were measured in the anterior hypothalamus (AH), medial hypothalamus (MH), pituitary gland and ovaries, whereas gonadotrophins and steroid hormones (i.e., progesterone, testosterone and estradiol) were measured in serum.

#### 2.3.2 Experiment 2: Administration of 5‐HTP into the ovarian bursa

This experiment was conducted to analyse the ovarian steroidogenic response to 5‐HTP administration. The 5‐HTP was micro‐injected into the ovarian bursa of rats (*n* = 8−10 per group). The treatment was conducted using previously described methods (Moran et al., 2013). Briefly, the animals were anaesthetized with sodium pentobarbitone (40 mg/kg body weight, i.p.; Anestesal; Smith Kline Norden of México, Monterrey, Nuevo León), placed on the surgery table in dorsal recumbency, and the right and left ovaries were exposed via a small incision made in the abdominal wall. In total, 1.5 µg of 5‐HTP dissolved in 40 µL of saline was injected into the bursa of each ovary using a 28‐gauge needle. The Vh group received the same quantity of saline via the same route of administration. To allow for diffusion of the 5‐HTP or saline into the ovarian bursa, the injection needle was held in the bursa for 1 min. After the injection, the ovaries were returned to the abdominal cavity, and the skin and muscle were sutured. The animals received an i.m. injection of meloxicam (Meloxi‐Jet; NorVet, SA DE CV Mexico, SAGARPA Q‐7827‐291) at a dose of 5 mg/kg body weight. Meloxicam is an analgesic and non‐steroidal anti‐inflammatory drug. After the surgery, each animal was placed in an individual recovery cage with artificial lighting. The dose of 5‐HTP injected into the ovarian bursa was calculated considering the dose of 100 mg/kg of 5‐HTP and the weight of the ovary (15 mg for the 30‐day‐old rats). In this experiment, neurotransmitters were measured in the ovaries, whereas steroid hormones were quantified in serum.

#### 2.3.3 Experiment 3: Effects of 5‐HTP micro‐injection into the MRN

For micro‐injection of 5‐HTP into the MRN, the rats (*n* = 8−10 per group) were anaesthetized with sodium pentobarbitone (40 mg/kg body weight, i.p.). Loss of the righting reflex following a tail pinch was considered an effect of anaesthesia (Goodchild et al., 2015). After confirming that the animals were adequately anaesthetized, they were placed in a stereotaxic apparatus (David Kopff Instruments, Tujunga, CA, USA). The skin of the head was opened, the skull drilled, and a number 29 needle (13 mm long) was introduced, following the parameters of the stereotaxic atlas (Paxinos & Watson, 2007). The coordinates for the MRN were anteroposterior = 0.5 mm from lambda, *lateral* = 4.9 mm at a 35° angle in relation to its perpendicular axis, and ventral = 8.0. The needle was connected via teflon tubing (0.65 mm outer diameter, 0.12 mm inner diameter; Bionalytical Systems, West Lafayette, IN, USA) to a Hamilton syringe mounted on a micro‐injection pump (CMA/100; Harvard Bioscience, Sweden). This was used to inject 20 µg of 5‐HTP dissolved in 2.5 µL of saline at an infusion rate of 1 µL/min. The Vh group received the same quantity of saline via the same route of administration. The animals received an i.m. injection of meloxicam (Meloxi‐Jet; NorVet) at a dose of 5 mg/kg body weight. After the surgery, each animal was placed in an individual recovery cage with artificial lighting. In this experiment, neurotransmitters were evaluated in the hypothalamus, whereas gonadotrophins were quantified in serum 60 or 120 min after micro‐injection of 5‐HTP.

The accuracy of the micro‐injection in the MRN was verified after dissection of the hypothalamus. The caudal portion of the brain of the treated rats was immersed in 10% formaldehyde for ≥48 h; subsequently, 100‐µm‐thick slices were obtained using a vibratome (Series 3000; Technical Products International, St Louis, MO, USA). Brain slices were stained with 1% Cresyl Violet. The location of the trajectory of the needle was observed histologically with the aid of a stereoscopic microscope. Only rats (*n* = 8−10 per group) with accurate micro‐injection in the MRN were included in the analysis.

### 2.4 Post‐mortem examination

All the animals were killed in a CO_2_ acrylic chamber measuring 40 cm wide × 40 cm long × 30 cm high at a fill rate of 30%–70% of the chamber volume per minute with CO_2_. The blood from the trunk was collected, allowed to clot, and centrifuged at 1000*g*. The serum was stored at −20°C until follicle‐stimulating hormone (FSH), LH, testosterone and estradiol levels were measured by radioimmunoassay. The ovaries and pituitary gland were dissected, weighed in a precision balance, and stored at −70°C for the subsequent quantification of 5‐HT, 5‐HIAA, DA and NA using high‐performance liquid chromatography (HPLC).

After careful sectioning of the nerves and optic chiasma, the brain was removed, placed in cold saline, frozen in liquid nitrogen, then placed on a cold metal plate with the dorsal side facing up. The AH and MH regions were dissected following the landmarks described in the stereotaxic atlas for rats (Paxinos & Watson, 2007) and a modification previously reported (Heffner et al., 1980). Then, using a set of three razor blades mounted in a customized cast aluminum cutting block, a pair of coronal slices were obtained from the brain. The first slice dissected the AH (bregma −0.8 mm to bregma 1.8 mm), which included the anterior and median preoptic area, the suprachiasmatic nucleus, the paraventricular nucleus, the periventricular nucleus and the AH. The second slice dissected the MH (bregma 2.3–3.3 mm), which included the median eminence and arcuate nucleus (Monroy et al., 2003). Both hypothalamic regions were stored at −70°C until analysis.

### 2.5 Evaluation of neurotransmitters

Measurement of 5‐HT, 5‐HIAA, DA and NA concentrations was performed according to previously described methods (Moran et al., 2013). In brief, the hypothalamic and ovarian samples were weighed and homogenized in 300 µL of 0.1 N perchloric acid, and the pituitary in 150 µL of 0.1 N perchloric acid, and subsequently centrifuged at 16 831*g* for 30 min at −4°C. The supernatant was filtered using 0.45 µm regenerated cellulose filters. Twenty microlitres of the extract were injected into the HPLC system for analysis. The HPLC apparatus consisted of an isocratic pump (model LC‐250; Perkin Elmer, Norwalk, CT, USA), a Rheodyne injection valve (20 µL) and a Biophase ODS C‐18 analytical column (25 cm × 4.6 mm, with 5 µm particle size; Bionalytical Systems, West Lafayette, IN, USA) protected by a precolumn ultrasphere ODS preanalytical column (3 cm × 4.6 mm, with 10 µm particle size; Bioanalytical Systems).

The concentrations of neurotransmitters were quantified electrochemically using a BAS LC‐4C amperometric detector and an LC‐4A glassy carbon transducer cell (Bioanalytical Systems). The mobile phase consisted of 0.1 M citrate buffer (pH 3.0), with 200 mg of 1‐octane‐sulfonic‐acid (Sigma), previously filtered, and degassed with helium in vacuum conditions. Twenty millilitres of acetonitrile and 21.5 mL of tetrahydrofuran (Omnisolve and Science, Canada) were immediately added. A total volume of 500 mL was used for chromatographic analysis. The phase was pumped at a flow rate of 1.2 mL/min. Stock standard (Sigma) solutions were diluted on the day of the experiment in 0.1 N perchloric acid. The system was calibrated from a standard curve obtained by injecting stock ranging in concentration from 0.1 to 1 ng/µL. 5‐Hydroxytryptamine was identified by the relative retention times compared with standards. Using a 1020 Perkin Elmer Nelson integrator, 5‐HT levels were determined by comparing high peak ratios of unknown samples with their respective standards. The sensitivity was 0.001 ng. Results were expressed as nanograms of neurotransmitter per milligram of wet tissue.

### 2.6 Evaluation of progesterone, testosterone and estradiol

Serum progesterone, testosterone and estradiol concentrations were measured by specific radioimmunoassay using a kit purchased from Diagnostic Products (Los Angeles, CA, USA). The intra‐ and interassay coefficients of variation were 5.3% and 9.87% for progesterone, 6.58% and 6.93% for testosterone, and 6.89% and 7.12% for estradiol, respectively. The detection limits for progesterone, testosterone and estradiol were 0.09 ng/mL, 2.0 pg/mL and 1.9 pg/mL, respectively.

### 2.7 Evaluation of gonadotrophins

The FSH and LH concentrations were measured by radioimmunoassay using the double antibody technique, with the reagents and protocol supplied by the National Hormone and Pituitary Program (Baltimore, MD, USA). For FSH, the antibody was NIDDK‐anti‐rFSH‐S‐11, and for LH the antibody was NIDDK‐anti‐rLH‐S‐11. The results were expressed in nanograms per millilitre as the international reference standards rLH‐RP‐3 and rFSH‐RP‐2. Intra‐ and interassay coefficients of variation for FSH were 5.74% and 7.91% and for LH they were 6.82% and 9.32%, respectively. The detection limits for FSH and LH were 0.01 and 0.05 ng/mL, respectively.

### 2.8 Statistics

Normality of the data for all variables measured in this study was checked by the Shapiro–Wilk test; whereas homoscedasticity was checked by Levene's test. When both these previous tests were non‐significant, one‐way ANOVA was used followed by Tukey's test. If Levene's test was significant but the Shapiro–Wilk test was not, one‐way ANOVA in a model of corrected for heterocedasticity followed by Tukey's test was applied. The Kruskal–Wallis test followed by Wilcoxon's test was applied when the following conditions were met: (1) the Shapiro–Wilk test was significant but Levene's test was not; and (2) the Shapiro–Wilk test and Levene's test were significant. For all tests, a *P*‐value of <0.05 was considered statistically significant. Results are presented as the mean ± SD. All analyses were performed using the software R v.4.2.2 and the library *car* v.3.1‐1.

## 3 RESULTS

In this study, the effects of administering 5‐HTP via different routes were compared against the Vh and AC groups. Owing to its physiological relevance, the effects of 5‐HTP administration against the Vh group are principally described below, although comparisons between 5‐HTP versus the AC group and between the Vh and AC groups were also performed. In some cases, the *n* was not 10 owing to the loss or unsuitability of the biological samples. In no case did the animals die owing to the treatment.

### 3.1 Experiment 1: Intraperitoneal injection of 5‐HTP

**FIGURE 2:**
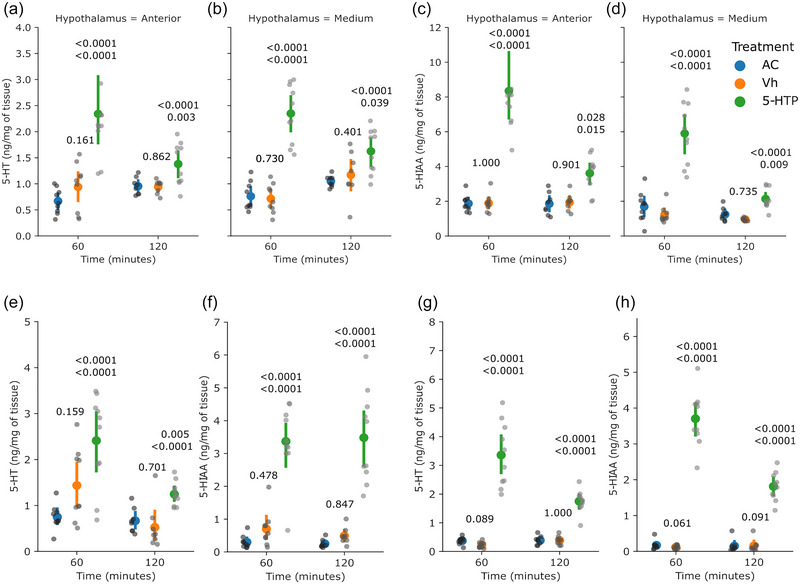
Systemic administration of 5‐HTP induced high values of the mean concentrations of 5‐HT and 5‐HIAA in the hypothalamus, hypophysis and ovaries. (a, b, e, g) Mean values of the concentrations of 5‐HT in the hypothalamus (a, b), hypophysis (e) and ovaries (g). (c, d, f, h) Concentrations of 5‐HIAA in hypothalamus (c, d), hypophysis (f) and ovaries (h) were also measured. The coloured dots and vertical lines indicate the mean and SD, respectively, by group. The grey dots are the individual values (one measurement per rat, 8–10 rats per group). The numbers above the 5‐HTP values are *P*‐values versus the AC and Vh groups (upper and lower, respectively); the number above the Vh values is the *P*‐value versus the AC group. When data were normally distributed and heterocedasticity was found, one‐way ANOVA in a model of orthogonal contrasts was applied. When data were not normally distributed, the Kruskal–Wallis test followed by Wilcoxon's test was applied (see Supplementary File 1). Abbreviations: AC, absolute control; 5‐HIAA, 5‐hydroxyindole‐3‐acetic acid; 5‐HT, 5‐hydroxytryptamine; 5‐HTP, 5‐hydroxytryptophan; Vh, vehicle.

For experiment 1, the rats (*n* = 8–10 per group) received an i.p. injection of 5‐HTP, which induced higher concentrations of 5‐HT in the AH (*P* < 0.0001 at 60 min and *P* = 0.003 at 120 min vs. Vh group; Figure 2a) and in the MH (*P* < 0.0001 at 60 min and *P* = 0.039 at 120 min vs. Vh group; Figure 2b). The concentrations of 5‐HIIA were also higher in the AH (*P* = < 0.0001 at 60 min and *P* = 0.015 at 120 min vs. Vh group; Figure 2c) and in the MH (*P* = < 0.0001 at 60 min and *P* < 0.009 at 120 min vs. Vh group; Figure 2d).

In the hypophysis, the measured concentrations of 5‐HT were higher (*P* < 0.0001 at 60 min and *P* = 0.0001 at 120 min vs. Vh group; Figure 2e), and the concentrations of 5‐HIIA were also higher (*P* < 0.0001 at 60 min and *P* < 0.0001 at 120 min; Figure 2f).

In the ovaries, the measured concentrations of 5‐HT were higher (*P* < 0.0001 at 60 min and *P* < 0.0001 at 120 min; Figure 2g), and the concentrations of 5‐HIIA were also higher (*P* < 0.0001 at 60 min and *P* < 0.0001 at 120 min; Figure 2h).

In general, 5‐HTP did not induce changes in the concentrations of NA nor DA. There were no significant differences in concentrations of NA in the AH at either 60 or 120 min (*P* = 0.294 and *P* = 0.648, respectively, vs. Vh group), nor in the MH at either 60 or 120 min (*P* = 0.216 and *P* = 0.969, respectively, vs. Vh group; Table 1). Noradrenaline was not detected in the hypophysis at 120 min and was unchanged at 60 min (*P* = 0.997 vs. Vh group), and in the ovaries it was not changed at either 60 or 120 min (*P* = 0.839 and *P* = 0.592, respectively, vs. Vh group). Dopamine was unchanged in the AH at either 60 or 120 min (*P* = 0.839 and *P* = 0.994, respectively, vs. Vh group) or in the MH at either 60 or 120 min (*P* = 0.576 and *P* = 0.995, respectively, vs. Vh group). In the hypophysis, dopamine was unchanged at either 60 or 120 min (*P* = 0.968 and *P* = 0.922, respectively, vs. Vh group), whereas in the ovaries, dopamine was undetectable.

**TABLE 1:** Effects of i.p. injection of 5‐hydroxytryptophan on concentrations of neurotransmitters (in nanograms per milligram of tissue) in the anterior or medial hypothalamus, pituitary and ovaries of female rats killed at different time points.

		Anterior hypothalamus		Median hypothalamus		Hypophysis		Ovaries	
Treatment	Time (min)	Noradrenaline	Dopamine	Noradrenaline	Dopamine	Noradrenaline	Dopamine	Noradrenaline	Dopamine
AC	60	1.43 ± 0.22	0.37 ± 0.51	1.64 ± 0.45	0.33 ± 0.10	0.53 ± 0.19	0.55 ± 0.17	0.32 ± 0.03	ND
Vh	60	1.77 ± 0.30 (0.157)	0.23 ± 0.09 (1.000)	1.59 ± 0.54 (0.964)	0.36 ± 0.18 (0.920)	0.49 ± 0.45 (0.979)	0.49 ± 0.28 (0.999)	0.22 ± 0.13 (0.671)	ND
5‐HTP	60	1.50 ± 0.54 (0.905, 0.294)	0.25 ± 0.10 (1.000, 0.839)	1.26 ± 0.17 (0.138, 0.219)	0.32 ± 0.10 (0.484, 0.576)	0.48 ± 0.31 (0.948, 0.997)	0.51 ± 0.17 (0.956, 0.968)	0.26 ± 0.19 (0.938 0.839)	ND
AC	120	0.65 ± 0.72	0.12 ± 0.15	0.42 ± 0.53	0.15 ± 0.18	1.72 ± 1.82	1.62 ± 2.98	0.32 ± 0.34	ND
Vh	120	0.49 ± 0.57 (0.964)	0.11 ± 0.12 (0.593)	0.36 ± 0.45 (0.856)	0.10 ± 0.15 (0.516)	ND	0.90 ± 1.22 (0.724)	0.33 ± 0.38 (1.000)	ND
5‐HTP	120	0.40 ± 0.54 (0.856, 0.648)	0.09 ± 0.13 (0.637, 0.994)	0.20 ± 0.32 (0.927, 0.999)	0.08 ± 0.14 (0.565, 0.995)	ND	0.44 ± 0.28 (0.492, 0.922)	0.20 ± 0.03 (0.592, 0.592)	ND

*Note*: Data are mean values ± SD. The mean of each parameter in each treatment and at each time point was calculated based on the results obtained from 8–10 rats per group, with one measurement per rat. The numbers in parentheses in the row for 5‐HTP represent *P*‐values; the first one corresponds to the comparison with AC, and the second one corresponds to the comparison with Vh at the corresponding time. The number in parentheses in the row for Vh represents the *P*‐value compared with the AC group.

Abbreviations: AC, absolute control; 5‐HTP, 5‐hydroxytryptophan; ND, not detectable (limit of detection is 0.0001 ng); Vh, vehicle.

**FIGURE 3:**
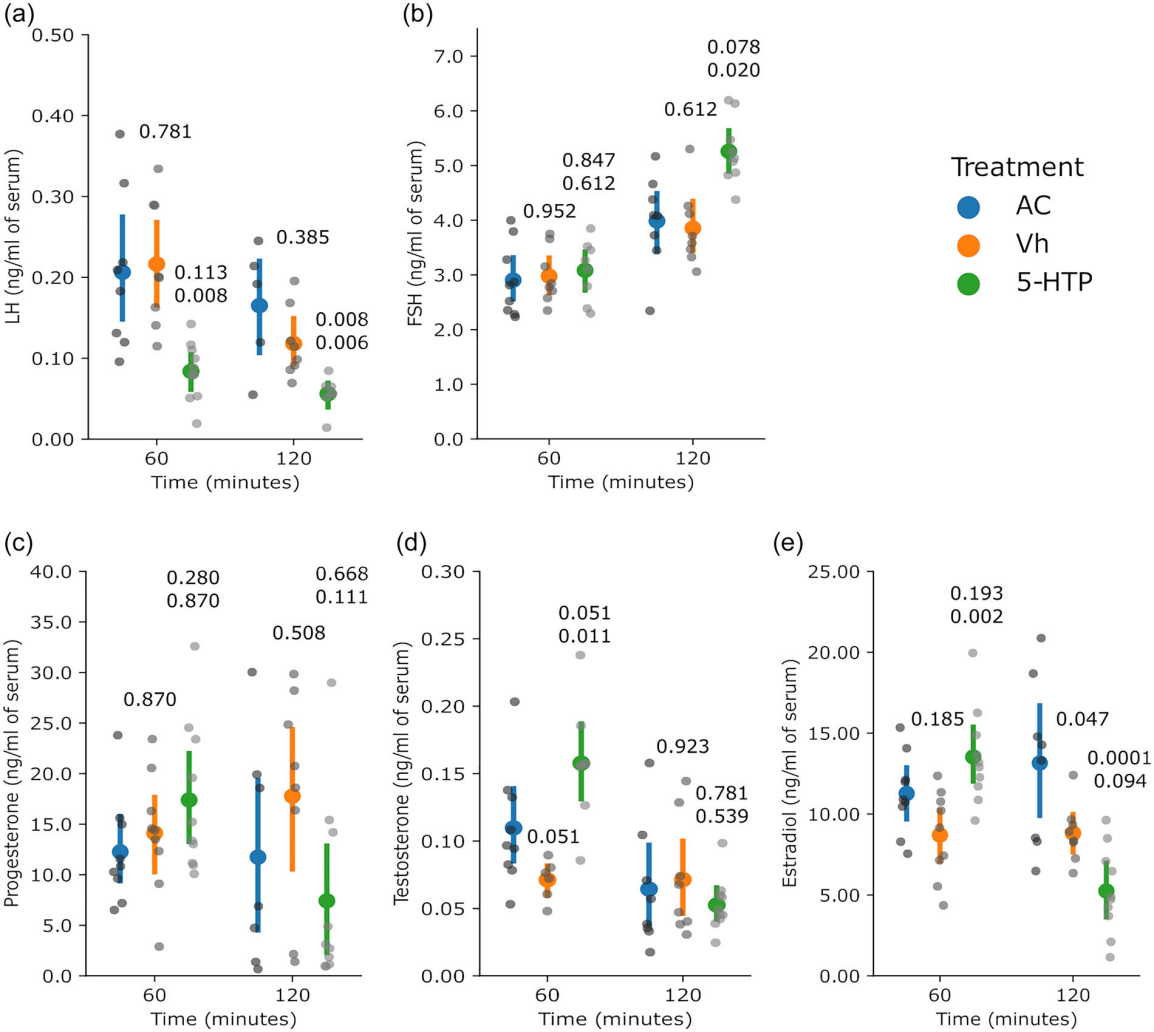
Systemic administration of 5‐HTP modified the mean values of circulating concentrations of gonadotrophins and sex steroids; the figure shows the mean values of the concentrations of LH (a), FSH (b), progesterone (c), testosterone (d) and estradiol (e). The coloured dots and vertical lines indicate the mean and SD, respectively, by group. The grey dots are the individual values (one measurement per rat, 8–10 rats per group). The numbers above the 5‐HTP values are *P*‐values versus the AC and Vh groups (upper and lower, respectively); the number above the Vh values is the *P*‐value versus the AC group. When data were normally distributed and heterocedasticity was found, one‐way ANOVA in a model of orthogonal contrasts was applied. When data were not normally distributed, the Kruskal–Wallis test followed by Wilcoxon's test was applied. Abbreviations: AC, absolute control; FSH, follicle stimulating hormone; 5‐HTP, 5‐hydroxytryptophan; LH, luteinizing hormone; Vh, vehicle.

**FIGURE 4:**
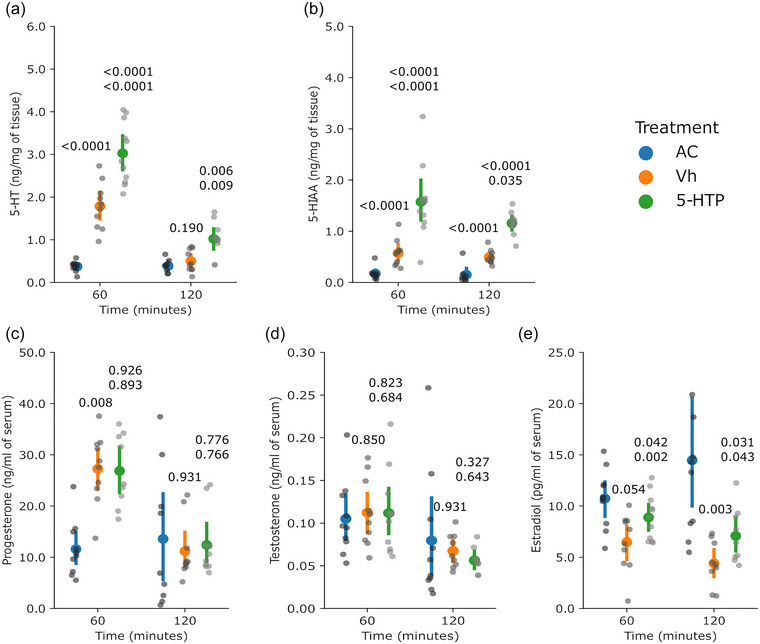
(a, b, e) Injection of 5‐HTP into the ovarian bursa increased the mean concentrations of ovarian 5‐HT (a) and 5‐HIAA (b), and circulating estradiol (e). (c, d) Also shown are mean values of the concentrations of progesterone (c) and testosterone (d). The coloured dots and vertical lines indicate the mean and SD, respectively, by group. The grey dots are individual values (one measurement per rat, 8–10 rats per group). The numbers above the 5‐HTP values are *P*‐values versus the AC and Vh groups (upper and lower, respectively); the number above the Vh values is the *P*‐value versus the AC group. When data were normally distributed and heterocedasticity was found, one‐way ANOVA in a model of orthogonal contrasts was applied. When data were not normally distributed, the Kruskal–Wallis test followed by Wilcoxon's test was applied. Abbreviations: AC, absolute control; 5‐HIAA, 5‐hydroxyindole‐3‐acetic acid; 5‐HT, 5‐hydroxytryptamine; 5‐HTP, 5‐hydroxytryptophan; Vh, vehicle.

The circulating concentrations of LH were lower (*P* = 0.008 at 60 min and *P* = 0.006 at 120 min vs. Vh group; Figure 3a) at both time points, whereas the concentrations of FSH were unchanged at 60 min (*P* = 0.612 vs. Vh group) but were higher after 120 min (*P* = 0.020 vs. Vh group; Figure 4b). The circulating concentrations of progesterone were not affected by the 5‐HTP treatment (*P* = 0.870 at 60 min and *P* = 0.111 at 120 min; Figure 3c), whereas the concentrations of testosterone and estradiol were higher at 60 min (*P* = 0.011 and *P* = 0.002, respectively; Figure 3d, e); however, concentrations of estradiol were higher at 60 min (*P* = 0.002 vs. Vh group) but unchanged at 120 min (*P* = 0.094; Figure 3e).

### 3.2 Experiment 2: Injection of 5‐HTP into the ovarian bursa

For experiment 2, the rats (*n* = 8–10 per group) received an injection of 5‐HTP into the ovarian bursa. The concentrations of 5‐HT measured in the ovary were higher (*P* < 0.0001 at 60 min and *P* = 0.009 at 120 min vs. Vh group; Figure 4a), and the concentrations of 5‐HIIA were higher (*P* < 0.0001 at 60 min and *P* = 0.035 at 120 min vs. Vh group; Figure 4b).

The concentrations of NA were not changed at either 60 or 120 min (*P* = 0.314 and *P* = 0.395, respectively, vs. Vh group). Dopamine was undetectable by the system at either 60 or 120 min (the limit of detection was 0.0001 ng; Table 2).

**TABLE 2:** Effects of injection of 5‐hydroxytryptophan into the ovarian bursa on concentrations of neurotransmitters (in nanograms per milligram of tissue) of female rats killed at different time points.

Treatment	Time (min)	Noradrenaline	Dopamine
AC	60	0.25 ± 0.32	0.05 ± 0.04
Vh	60	0.18 ± 0.06 (0.246)	ND
5‐HTP	60	0.16 ± 0.06 (0.059, 0.314)	ND
AC	120	0.33 ± 1.02	ND
Vh	120	0.10 ± 0.03 (0.017)	0.07 ± 0.03
5‐HTP	120	0.19 ± 0.16 (0.395, 0.395)	ND

*Note*: Data are mean values ± SD. The mean of each parameter in each treatment and at each time point was calculated based on the results obtained from 8–10 rats per group, with one measurement per rat. The numbers in parentheses in the row for 5‐HTP represent *P*‐values; the first one corresponds to the comparison with AC, and the second one corresponds to the comparison with Vh at the corresponding time. The number in parentheses in the row for Vh represents the *P*‐value compared with the AC group.

Abbreviations: AC, absolute control; 5‐HTP, 5‐hydroxytryptophan; ND, not detectable (limit of detection is 0.0001 ng); Vh, vehicle.

Administration of 5‐HTP had no effect on the circulating concentrations of progesterone (*P* = 0.893 at 60 min and *P* = 0.766 at 120 min vs. Vh group; Figure 4c) and testosterone (*P* = 0.684 at 60 min and *P* = 0.643 at 120 min vs. Vh, group; Figure 4d), but induced higher values of estradiol (*P* = 0.002 at 60 min and *P* = 0.043 at 120 min vs. Vh group; Figure 4e).

### 3.3 Experiment 3: Injection of 5‐HTP into the MRN

**FIGURE 5:**
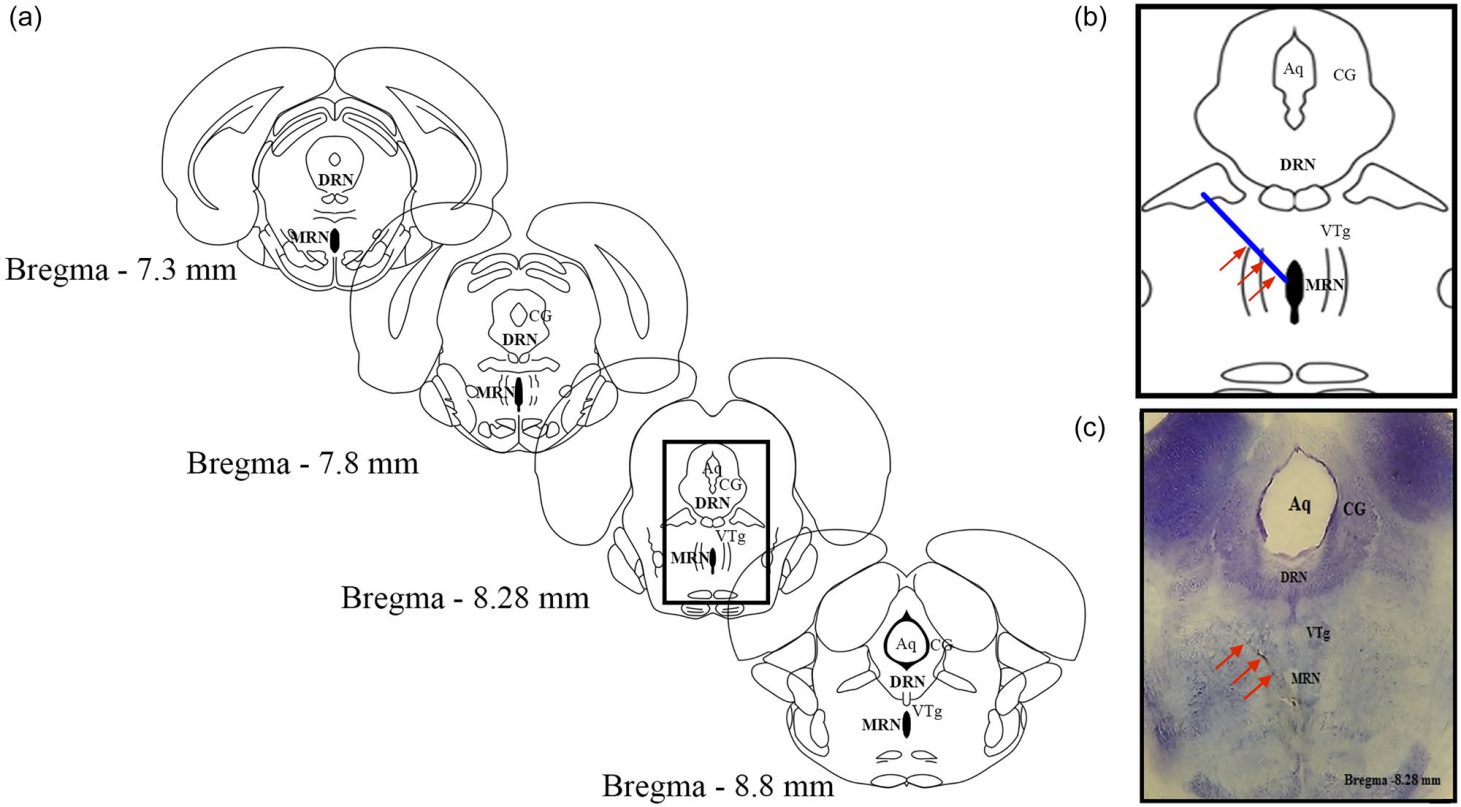
Rats with a successful injection of 5‐hydroxytryptophan into the median raphe were included in the analysis. (a) The anatomical location of the DRN is represented schematically; the injection site is enclosed in a rectangle. (b) The injection site is magnified to show the trajectory of the microneedle into the MRN (blue line emphasized by red arrows). (c) A Nissl‐stained coronal section illustrates the trajectory of the microneedle into the MRN; the red arrows indicate the trajectory of the micro‐injection into the MRN. Abbreviations: Aq, cerebral aqueduct; CG, central grey; DRN, dorsal raphe nucleus; MRN, median raphe nucleus; VTg, ventral tegmental nucleus.

**FIGURE 6:**
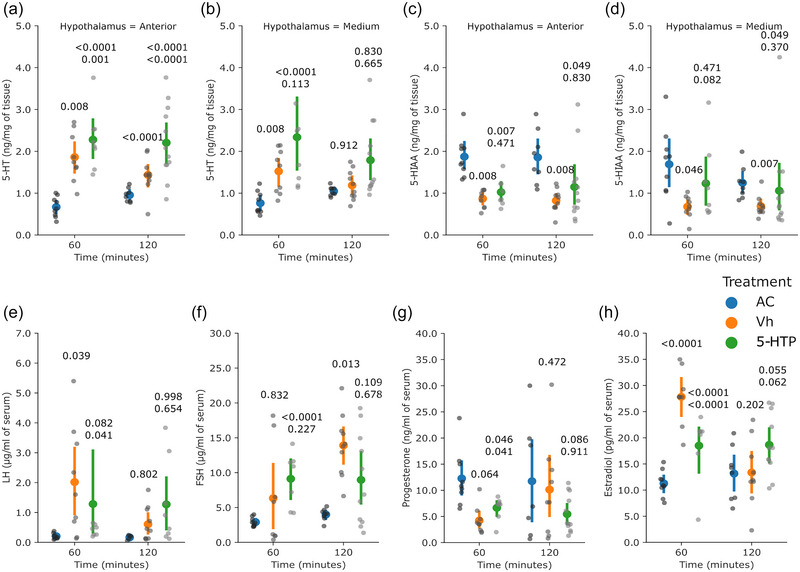
Injection of 5‐HTP into the MRN did not change the mean concentrations of ovarian 5‐HT and 5‐HIAA nor circulating concentrations of gonadotrophins but had an impact on concentrations of estradiol. The figure shows concentrations of 5‐HT (a, b) and 5‐HIAA (c, d) in the hypothalamus, and circulating LH (e), FSH (f), progesterone (g) and estradiol (h). The coloured dots and vertical lines indicate the mean and SD, respectively, by group. The grey dots are the individual values (one measurement per rat, 8–10 rats per group). The numbers above the 5‐HTP values are *P*‐values versus the AC and Vh groups (upper and lower, respectively); the number above the Vh values is the *P*‐value versus the AC group. When data were normally distributed and heterocedasticity was found, one‐way ANOVA in a model of orthogonal contrasts was applied. When data were not normally distributed, the Kruskal–Wallis test followed by Wilcoxon's test was applied. Abbreviations: AC, absolute control; FSH, follicle stimulating hormone; 5‐HIAA, 5‐hydroxyindole‐3‐acetic acid; 5‐HT, 5‐hydroxytryptamine; 5‐HTP, 5‐hydroxytryptophan; LH, luteinizing hormone; MRN, median raphe nucleus; Vh, vehicle.

For experiment 3, only the rats with accurate micro‐injection in the MRN were included in the study (Figure 5). The rats (*n* = 8–10 per group) received a micro‐injection of 5‐HTP into the MRN, which induced higher concentrations of 5‐HT in the AH at either 60 or 120 min (*P* = 0.001 and *P* < 0.0001, respectively, vs. Vh group; Figure 6a), but no changes were observed in the MH (*P* = 0.471 at 60 min and *P* = 0.830 at 120 min vs. Vh group; Figure 6b). The concentrations of 5‐HIAA were also unchanged in the AH (*P* = 0.471 at 60 min and *P* = 0.830 at 120 min; Figure 6c) and in the MH (*P* = 0.082 at 60 min and *P* = 0.370 at 120 min, respectively, vs. Vh group; Figure 6d).

In the AH, the concentrations of NA were unchanged at either 60 or 120 min (*P* = 1.000 and *P* = 0.754, respectively, vs. Vh group), whereas in the MH, the concentrations were lower at 60 min (*P* = 0.012 vs. Vh group) but were unchanged after 120 min (*P* = 0.960 vs. Vh group; Table 3). The concentrations of DA were undetectable in the MH of the Vh group after 120 min; however, they were unchanged after 60 min in the AH (*P* = 0.770 vs. Vh group) and MH (*P* = 1.000 vs. Vh group) and after 120 min (*P* = 1.000) in the AH (Table 3).

**TABLE 3:** Effects of micro‐injection of 5‐hydroxytryptophan in the median raphe on neurotransmitters (in nanograms per milligram of tissue) of female rats killed at different time points.

		Anterior hypothalamus		Medium hypothalamus	
Treatment	Time (min)	Noradrenaline	Dopamine	Noradrenaline	Dopamine
AC	60	1.43 ± 0.22	0.37 ± 0.51	1.64 ± 0.45	0.33 ± 0.10
Vh	60	1.50 ± 0.87 (0.067)	0.30 ± 0.15 (0.670)	2.19 ± 0.42	0.53 ± 0.30 (1.000)
5‐HTP	60	1.56 ± 0.31 (1.000, 1.000)	0.34 ± 0.11 (0.220, 0.770)	1.71 ± 0.51 (0.119, 0.012)	0.36 ± 0.07 (1.000, 1.000)
AC	120	0.65 ± 0.72	0.12 ± 0.15	0.42 ± 0.53	0.15 ± 0.18
Vh	120	1.53 ± 0.72 (1.000)	0.43 ± 0.30 (1.000)	2.31 ± 1.20 (0.160)	ND
5‐HTP	120	1.63 ± 0.62 (1.000, 0.754)	0.51 ± 0.37 (1.000, 1.000)	1.79 ± 0.48 (0.360, 0.960)	0.70 ± 0.34 (0.011)

*Note*: Data are mean values ± SD. The mean of each parameter in each treatment and at each time point was calculated based on the results obtained from 8–10 rats per group, with one measurement per rat. The numbers in parentheses in the row for 5‐HTP represent *P*‐values; the first one corresponds to the comparison with AC, and the second one corresponds to the comparison with Vh at the corresponding time. The number in parentheses in the row for Vh represents the *P*‐value compared with the AC group.

Abbreviations: AC, absolute control; 5‐HTP, 5‐hydroxytryptophan; ND, not detectable (limit of detection is 0.0001 ng); Vh, vehicle.

The circulating concentrations of LH were lower at 60 min (*P* = 0.041 vs. Vh group) but unchanged at 120 min (*P* = 0.654 vs. Vh group; Figure 6e), whereas the concentrations of FSH were unchanged (*P* = 0.227 at 60 min and *P* = 0.678 at 120 min vs. Vh group; Figure 6f). The circulating concentrations of progesterone were higher at 60 min (*P* = 0.041 vs. Vh group) but unchanged at 120 min (*P* = 0.911 vs. Vh group; Figure 6g). In this experiment, the concentrations of testosterone were evaluated, but for 90% of the samples the levels of the hormone were below the limit of detection, hence there were insufficient data to for analysis. The circulating concentrations of estradiol were lower at 60 min (*P* < 0.0001) but unchanged at 120 min (*P* = 0.062; Figure 6h).

## 4 DISCUSSION

The results obtained in this study suggest that in prepubertal female rats, activation of the serotonergic system induced by the administration of 5‐HTP is accompanied by deregulation of gonadotrophin production by the pituitary gland and an increase in estradiol production by the ovary. This effect is related to the route of administration.

Administration of the vehicle induced changes in some of the parameters evaluated; these changes were marked with either direct injection into the ovarian bursa or micro‐injection into the MRN, but not by the i.p. route. The effects of the vehicle are possibly attributable to stress and the activation of the hypothalamic–hypophysial–adrenal axis (Freiman et al., 2016), induced by the surgical manipulation or by the anaesthesia. However, it is unlikely that these changes are solely attributable to stress. Another contributory factor could be the modification of neural pathways that connect to or originate from the NRM, which regulate ovarian functions.

The lack of modification in the concentrations of NA and DA suggests that the effects of 5‐HTP administration are specific to the serotonergic system. Our results showed that 5‐HTP increased the synthesis of 5‐HT in the hypothalamic–hypophysial–ovarian axis, which resulted in changes in testosterone and estradiol production in the ovary. In the CNS, there is a relationship between 5‐HT and 5‐HIAA concentrations (Kerdelhué et al., 1989). In this work, we observed an increase in the concentrations of 5‐HT and 5‐HIAA, in the AH and MH, the hypophysis and the ovaries after 5‐HTP injection. This stimulatory effect is evident in the three components of the reproductive axis (hypothalamus, hypophysis and ovary) when 5‐HTP is administered i.p. or into the ovarian bursa. Our findings could help to explain the results of previous reports, in which the systemic administration of tryptophan increased the concentrations of 5‐HT in the hypothalamus (Justo et al., 1989; Petersen et al., 1989; Ruddick et al., 2006) and cerebrospinal fluid (Young & Teff, 1989).

In contrast to observations in the hypothalamus of animals after i.p. injection of 5‐HTP, when the micro‐injection was performed directly into the MRN, the concentrations of 5‐HT and 5‐HIAA in the hypothalamus were unchanged. When i.p. injection was performed, 5‐HTP diffused to all neurons that comprise the DRN and MRN (11 500 and 1100, respectively; Jacobs & Azmitia, 1992) and that innervate the hypothalamus. An explanation for the distinct effects of 5‐HTP as a function of the route of administration is related to the quantity of neurons exposed. When micro‐injection was performed directly into the MRN, only the serotonergic neurons of this nucleus, which represent 8.73% of the forebrain‐projecting 5‐HT neurons (DRN + MRN 5‐HT cells), were exposed to the 5‐HTP. The low percentage of neurons exposed could not be sufficient to induce a change in the concentration of 5‐HT in the hypothalamus after 60 min of treatment.

The increased concentrations of 5‐HT and 5‐HIAA in the ovary after the administration of 5‐HTP i.p. or directly into the ovarian bursa are explained by the presence of enzymes involved in the synthesis and metabolism of 5‐HT. Expression of the enzyme TPH1 has been described in the ovarian follicle (Nikishin et al., 2018), whereas the specific cellular types expressing the serotonergic enzyme aromatic amino acid decarboxylase have been reported in granulosa, theca and mast cells (Nikishin et al., 2019).

The effects of 5‐HTP on gonadotrophin secretion vary depending on the route of administration. The i.p. injection of 5‐HTP increased 5‐HT concentrations in the hypothalamus at 60 and 120 min. This increase coincided with lower serum LH concentrations, suggesting that in 30‐day‐old prepubertal rats, 5‐HT inhibits LH secretion, as previously reported (Arias et al., 1990), whereas FSH levels rose at 120 min. This differential effect of 5‐HT on gonadotrophin secretion might be related to the expression of 5‐HT receptor subtypes. During the prepubertal development of mice, changes in the expression of serotonin receptor subtypes have been observed. In the prepubertal female mouse hypothalamus (postnatal weeks 3, 4 and 8), there is a decrease in the expression of the 5‐HT_1a_ receptor, whereas 5‐HT_2c_, 5‐HT_4_ and 5‐HT_7_ receptors increase (Zhou et al., 2021). Additionally, Bhattarai et al. (2014) suggested that in prepubertal female mice, the 5‐HT pathway, through its 5‐HT_1a_ and 5‐HT_2c_ receptors, differentially regulates the excitability of GnRH neurons. The balance in the expression of these receptors either inhibits or activates them, consequently impacting the secretion of gonadotrophins. In 21‐day‐old prepubertal female rats, gonadotrophin secretion is stimulated via activation of 5‐HT_1_ and 5‐HT_3_ receptors but not by the 5‐HT_2_ receptor (Lacau‐Mengido et al., 1996). However, this evidence does not fully explain the differences in FSH and LH secretion observed in animals after i.p. injection of 5‐HTP. Here, we propose that in 30‐day‐old female rats: (1) the 5‐HT receptor subtypes 5‐HT_2b_, 5‐HT_4_, 5‐HT_6_ and 5‐HT_7_ might mediate the inhibitory effects of 5‐HT on LH secretion; and (2) the number or function of 5‐HT receptor subtypes changes during prepubertal development, contributing to the differential effect of 5‐HT on gonadotrophin secretion. This proposal supports the idea that 5‐HT activates different receptor subtypes, thereby differentially modulating gonadotrophin secretion depending on the specific 5‐HT receptor expressed in the hypothalamus.

Unlike the results observed with i.p. 5‐HT injection, when 5‐HTP was micro‐injected directly into the MRN, the decrease in LH concentration at 60 min can be attributed to increased 5‐HT concentrations in the AH at the same time point. Furthermore, in female adult rats, electrical stimulation of the MRN has been shown to reduce LH concentrations (Morello et al., 1989, 1990). These findings collectively support the idea that serotonergic innervation of the hypothalamus, originating in the MRN, exerts an inhibitory influence on the regulation of LH secretion.

The increase in progesterone seen in animals with 5‐HTP micro‐injection into the MRN is confusing. Luteinizing hormone typically stimulates ovarian progesterone production (Hu et al., 2010), but in these animals the LH levels dropped. One possible explanation is that the progesterone surge results from stress caused by the surgical procedure. This aligns with previous research showing that both prepubertal and adult rats respond to stress by releasing more progesterone from the adrenal gland (Romeo et al., 2004). Additionally, it is worth noting that 30‐day‐old female rats lack luteal bodies, the primary source of ovarian progesterone (Picut et al., 2015). Therefore, we propose that the increased progesterone observed in animals with MRN 5‐HTP micro‐injection is likely to originate from the adrenal gland, activated owing to stress. In contrast, when 5‐HTP was administered i.p., there were no noticeable changes in progesterone concentrations. This suggests that the rapid 5‐HTP injection procedure might not have been sufficiently stressful to activate the adrenal gland and result in a short‐term increase in progesterone concentrations. In support of this idea, it has been suggested that the hypothalamic–hypophysial–adrenal axis response varies according to the duration or nature of the stressor used (Ariza Traslaviña et al., 2014).

Given that the increases in testosterone and estradiol concentration observed in animals injected i.p. with 5‐HTP were not accompanied by increased concentrations of LH and FSH, we propose that these changes are not associated with modifications of the secretion of gonadotrophins. Luteinizing hormone in the theca cells of the ovarian follicle stimulates the biotransformation of cholesterol into pregnenolone, which forms progesterone (Handa & Weiser, 2014). This result indicates that intra‐ovarian factors, such as 5‐HT, are involved in the regulation of steroidogenesis in the gonad. The increase in 5‐HT concentration in the ovary and estradiol in the serum of animals after i.p. injection of 5‐HTP or injection into the ovarian bursa supports this idea. The possibility that intra‐ovarian 5‐HT regulates steroidogenesis is supported by evidence suggesting that: (1) granulosa cells synthesize 5‐HT (Dubé & Amireault, 2007) and transform the androgens to oestrogens (Hu et al., 2010); and (2) 5‐HT synthesized by mast cells is incorporated into the oocyte and follicular cells by the specific serotonin transporter protein (SERT), which is expressed in the granulosa cells and the ovocyte in the follicle (Nikishin et al., 2019).

95‐Hydroxytryptamine regulates the secretion of steroid hormones by modulating the activity of enzymes that modulate the synthesis of these hormones. The stimulatory effect on the synthesis of steroid hormones when 5‐HTP was administered into the ovarian bursa is in line with evidence from pre‐ovulatory follicles from adult rats, cultured in vitro. When ketanserin, an inhibitor of 5‐HT_2_ receptors, is present in culture medium for the follicles, production of progesterone and testosterone decreases, and this effect is reversed by addition of pregnenolone (Tanaka et al., 1993). In contrast, addition of 5‐HT to the medium modifies expression of the protein that mobilizes cholesterol to the mitochondria, the steroidogenic acute regulatory protein (StAR), and the production of estradiol (Yang et al., 2015).

Androgens are aromatized to estrogens in granulosa cells (Handa & Weiser, 2014); therefore, the stimulatory effect of 5‐HT on the synthesis of estradiol 60 min after injection of 5‐HTP into the ovarian bursa could be attributable to activation of the aromatase enzyme, which transforms testosterone into estradiol. This idea is supported by the fact that in mice that do nz<ot express the 5‐HT transporter, activity of the aromatase and the levels of estradiol in the serum decreased (Zha et al., 2017). It is not clear why there is an inverse effect on the estradiol concentrations, 120 min after administration 5‐HTP; however, the low concentrations of testosterone might have been insufficient to maintain the elevated levels of estradiol.

In this work, we showed that stimulation of the serotonergic system of the reproductive axis increased secretion of estradiol by the ovary. Exposure of females to high levels of oestrogens might have implications for the onset of sexual maturation and for the health of women, because estradiol not only regulates reproduction, but is also implicated in the control of several physiological processes involving the cardiovascular, skeletal, neuroendocrine and immune systems and other biological processes, such as cognition and behaviour (Pillerová et al., 2021). Also, increased estradiol production is associated with the development of several diseases, such as breast and ovarian cancers (Brown & Hankinson, 2015).

Based on the present results, we demonstrate that systemic administration of 5‐HTP increases 5‐HT concentrations in the reproductive axis components (hypothalamus, pituitary and ovaries), and in the gonad when locally administered in the ovarian bursa. In response to this increase, estradiol synthesis is activated in the ovaries. This increase is not accompanied by parallel changes in gonadotrophin production by the pituitary.

The above findings are relevant, because increasing the 5‐HT concentration in the hypothalamus by administration of 5‐HTP could be beneficial in the treatment of behavioural disorders, such as depression and sleep disorders in children and adolescents, as reported by some authors (Bruni et al., 2004; Cross et al., 2011; Ryan et al., 1992). However, its effects on gonadal physiology, evaluated through estradiol synthesis, have not yet been demonstrated. This is an important aspect to consider because, during this stage of development, oestrogens, among other factors, regulate the sexual development of women and the acquisition of their reproductive capacity.

## AUTHOR CONTRIBUTIONS

María E. Ayala, Andrés Aragón and Juana Monroy planned the experiments. Omar D. Cortés, Eloir Gallegos, María Eugenia Mendoza‐Garrido and Mario Cárdenas performed the experiments. María E. Ayala, Juana Monroy, Andrés Aragón and Roberto Domínguez participated in the analysis and discussion of the results. All authors have read and approved the final version of this manuscript and agree to be accountable for all aspects of the work in ensuring that questions related to the accuracy or integrity of any part of the work are appropriately investigated and resolved. All persons designated as authors qualify for authorship, and all those who qualify for authorship are listed.

## CONFLICT OF INTEREST

The authors declare no conflicts of interest.

## Data Availability

All data of this work are available on reasonable request.
